# Reduced Graphene Oxide-Silver Nanoparticles for Optical Pulse Generation in Ytterbium- and Erbium-Doped Fiber Lasers

**DOI:** 10.1038/s41598-020-66253-w

**Published:** 2020-06-10

**Authors:** Harith Ahmad, Hissah Saedoon Albaqawi, Norazriena Yusoff, Siti Aisyah Reduan, Chong Wu Yi

**Affiliations:** 1 Photonics Research Centre, University of Malaya, 50603 Kuala Lumpur, Malaysia; 2 Department of Physics, Faculty of Science, University of Malaya, 50603 Kuala Lumpur, Malaysia

**Keywords:** Optics and photonics, Physics

## Abstract

This work has demonstrated the potential of a reduced graphene oxide silver/polyvinyl alcohol (rGO-Ag/PVA) film as a saturable absorber (SA) in ytterbium and erbium based Q-switched optical fiber lasers. The facile hydrothermal method was used to synthesize the nanocomposite between rGO and Ag nanoparticles. This was followed by a simple solution method to form the rGO-Ag film using PVA as the host polymer. From nonlinear absorption characterization, the rGO-Ag/PVA SA was determined to have a modulation depth of 30%, a nonsaturable loss of 70%, and a saturable intensity of 0.63 kW/cm^2^. Stable self-starting Q-switched pulses were obtained at the threshold pump power of 72.76 mW and 18.63 mW in the ytterbium-doped (YDFL) and erbium-doped fiber laser (EDFL) cavities respectively. The center operating wavelengths were observed at 1044.4 nm and 1560 nm for the two cavities. The shortest pulse width and maximum repetition rate of the YDFL and EDFL were 1.10 µs and 62.10 kHz and 1.38 µs and 76.63 kHz respectively. This work has demonstrated that the rGO-Ag/PVA film is suitable as an SA for pulse generation in the 1.0 and 1.5 μm regions and would have many potential photonics applications.

## Introduction

In general, pulsed laser can be operated in Q-switching or mode-locking mode. Q-switching is obtained by modulating the intracavity losses and gives short pulses with high energy and peak power. Whereas, in the case of mode-locking, oscillating multiple longitudinal modes are phased locked as to generate ultrafast pulses in the picosecond or femtosecond durations. Mode-locking in a fiber laser can be achieved actively or passively^1^. The active mode-locking is normally achieved by periodically modulating the resonator losses or the round-trip phase change. This can be done using an acousto-optic, electro-optic, Mach–Zehnder integrated-optic or a semiconductor electro absorption modulator which is often placed at one end of the resonator. In the case of passive mode-locking, the generation of short pulses can be achieved using a saturable absorber (SA) as to modulate the resonator losses, thereby producing shorter pulses ^2,3^. Materials with two dimensional (2D) layered structures^4^ and ultra-small quantum dots^5–7^ have been successfully demonstrated to generate passively mode-locked pulses. Mode-locking and Q-switching operation have their own advantages towards specific application^8–10^.

Q-switched optical fiber lasers have received significant interest over the past decade due to their ability to generate pulses with higher pulse energies and over longer durations^11^. These characteristics make Q-switched optical fiber lasers ideal for various applications such as material processing^12^, remote sensing^13^ and range finding^14^ as well as finding numerous applications in medicine^15^. Typically, Q-switched pulses were obtained through active means, using acousto-optic^16^ or electro-optic modulators^17^. However, the bulk and cost of these modulators had spurred researchers to seek other Q-switching mechanisms that would be suitable for real-world applications. The solution to this was found in the form of passive Q-switching using saturable absorber (SAs). Passively Q-switched optical fiber lasers had many inherent advantages over the active counterparts, including simple and flexible design, cost effectiveness and requiring only minimal power supplies to operate.

In this regard, two dimensional (2D) materials such as black phosphorus^18^, graphene^19^, transition metal dichalcogenides (TMD)^20,21^, topological insulator (TI)^22,23^, and MAX phase materials^24^ has become widely used as SAs for passive Q-switching. The impressive optical properties of 2D materials that include a high modulation depth, short recovery time, broadband absorption, and low saturation fluence have make them suitable candidates as SAs for various lasers operating at different wavelengths^25^. By taking the advantage of their nonlinear optical responses, 2D materials can also been applied to optical devices such as optical modulator^26^ and optical switches^27^. Being one of the most attractive 2D materials graphene possesses several merits that make it suitable to be used as an SA having a wide spectral range and ultrafast recovery time for low-cost light modulation techniques^28^. Reduced graphene oxide (rGO) has also attracted much attention due to its optical properties such as saturable absorption ability and optical nonlinearity^29^. Furthermore, rGO is much easier and cost effective to fabricate as compared to pure graphene. Previous reports have shown that reduced graphene oxide (rGO) prepared by the thermal reduction of graphene oxide (GO) on fluorine mica (FM) can be utilized as SAs for Q-switched pulse generation in EDFLs^30^.

Furthermore, metal nanoparticles such as silver (Ag) and gold (Au) have also been demonstrated as potential SAs^31,32^. Ag nanoparticles have inherent advantages such as large third-order nonlinearity, broadband surface plasmon resonance (SPR) absorption and fast response time^33^. The unique linear and nonlinear optical properties of Ag nanomaterials arise due to their surface plasmons whereby the percentage of the surface atoms are significantly higher as compared to that of their bulk material counterparts^34^. Recently, Lokman *et al*.^35^ reported a Q-switched EDFL with a pulse energy of 146.4 nJ, with an average power of 20.5 mW at a pump power of 90.4 mW using silver nanoparticles (AgNP) as an SA. Inspired by the positive development of rGO and Ag nanoparticles as potential materials for SA due their unique properties, it will be interesting to investigate the performance of a new composite material consisting of rGO and Ag nanoparticles as an SA. Moreover, the absorption properties of the rGO-Ag composite, in particular its nonlinear absorption performance is still unknown and thus warrants this study. It is expected that the new composite material will demonstrate significant SA properties due to the complementary characteristics of the rGO and Ag nanoparticles.

In this paper, a passively Q-switched YDFL and EDFL using an rGO-Ag/PVA film as the SA was proposed and its operation demonstrated. The rGO-Ag nanocomposites were prepared using the facile hydrothermal method, which is a cost effective and reproducible technique for producing graphene-based nanocomposites. Using the rGO-Ag/PVA SA, the YDFL and EDFL cavities are able to produce pulses with energies of 3.52 nJ and 44.0 nJ and durations of 1.10 µs and 1.38 µs respectively at the maximum pump power. The maximum repetition rates of the YDFL and EDFL were 62.10 kHz and 76.63 kHz. The incorporation of the Ag nanoparticles into the rGO based SA has enhanced the nonlinear absorption characteristics as well as improved the overall laser performance. To the best of author’s knowledge, there have been no works reported on the use of rGO-Ag/PVA films as SAs for Q-switched YDFLs and EDFLs.

## Characterization of rGO-Ag Nanocomposite

**Figure 1:**
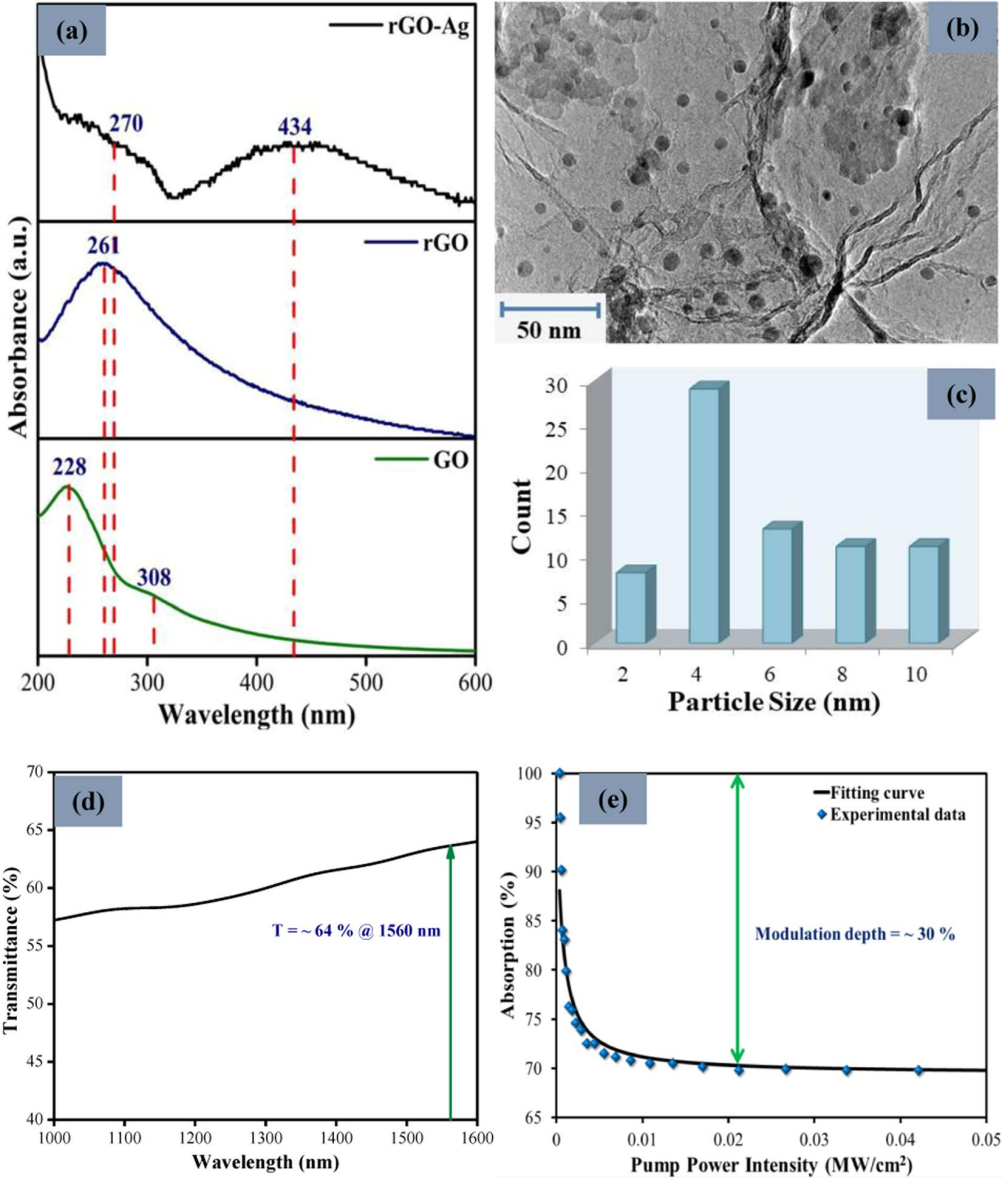
(**a**) UV-vis spectrum of GO, rGO, and rGO-Ag nanocomposite. (**b**) HRTEM image of rGO-Ag nanocomposite along with (**c**) the AgNPs particle size histogram. (**d**) Linear transmission and (**e**) nonlinear absorption spectrum of rGO-Ag/PVA SA.

The UV-Visible (UV-Vis) absorption spectrum of rGO, Ag nanoparticles (AgNPs), and rGO-Ag nanocomposite were obtained with an Agilent Technologies Varian Cary 50 UV–Vis Spectrophotometer, scanned from 200 to 600 nm. As displayed in Fig. 1(a), an absorption peak with maxima at 228 nm can be observed from the UV-Vis spectrum of GO which corresponds to the π → π* transitions of aromatic C=C bonds. A small bump located at 308 nm can also be detected due to the π → π* transition of C=O bonds (carbonyl groups)^36^. After the hydrothermal treatment, the absorption peak at 228 nm of the GO has shifted to 261 nm in the UV-vis spectrum of the rGO sample, which clearly suggests that the C=C bonds have been restored. Meanwhile, the disappearance of the peak at 308 nm was due to the reduction of C=O functional group which indicate that the reduction of GO has occurred, thus forming the rGO^37^. Moreover, the UV-Vis spectrum of the rGO-Ag nanocomposite shows two absorption peaks located at 270 and 434 nm, which are the characteristics of rGO and the AgNPs. The AgNPs absorption peak at 434 nm arises due to the formation of dipole plasmon resonance of spherical nanoparticles^38^, which is evidence of the formation of rGO-Ag nanocomposites after the hydrothermal treatment. The high-resolution transmission electron microscope (HRTEM) image of the rGO-Ag nanocomposites were recorded using a JEOL HRTEM model JEM 2100-F with an accelerating voltage of 200 kV. Figure 1(b) shows the HRTEM image of the rGO-Ag nanocomposites while the particle size distribution of AgNPs on the rGO surface is given as a histogram in Fig. 1(c). The rGO possess a sheet-like structure with some ‘wrinkles’ on the surface as a result of extended surface defects from the folding and twisting of the few layer rGO sheets^39^. It can be seen that the AgNPs which are spherical in shape with sizes ranging from 2 to 10 nm in diameter on the surface of the rGO sheet, confirming the successful synthesis of the rGO-Ag nanocomposite.

The linear transmission of the rGO-Ag/PVA SA was investigated by connecting a white light source to the SA assembly which was form using two fiber ferrules with the rGO-Ag/PVA film sandwiched in between and the spectrum recorded using an optical spectrum analyzer (OSA). As can be seen in Fig. 1(d), the rGO-Ag/PVA SA demonstrated a transmission of about 64% at 1560 nm. The balanced twin detection method was conducted to study the intensity-dependent absorbance of the rGO-Ag/PVA film where a Menlo Systems ELMO femtosecond erbium laser was employed as the incident optical pulse source. The laser source has a central wavelength at 1564 nm with the repetition rate and pulse width of 100 MHz and 2.88 ps, respectively. A variable optical attenuator (VOA) was connected to the laser source followed by a 50:50 coupler. The coupler was used to divide the source light into two parts and the VOA was utilized to tune the power of light passing through coupler. One arm of the coupler was attached to the power meter for reference while another arm was connected to the SA and then to a second power meter. The ratio of the two power channels will give necessary results of the saturable absorption. The nonlinear optical absorption characteristics of the rGO-Ag/PVA SA is shown in Fig. 1(e). The saturation model equation^40^ is given as:

$$\alpha (I)=\frac{{\alpha }_{s}}{1+I/{I}_{sat}}+{\alpha }_{ns}$$

where *α*_*s*_ is the modulation depth, *I* is the input intensity, *I*_*sat*_ is the saturation intensity and *α*_*ns*_ is the non-saturable loss. Fitting the experimental data to the above equation gives the modulation depth, saturation intensity and non-saturable loss of the SA which is approximately 30%, 0.63 kW/cm^2^ and 70% respectively. It should be noted that the modulation depth attained in this work was comparable to reported Q-switched pulsed lasers using other 2D materials as SAs^41,42^. This indicates that the modulation depth of the rGO-Ag/PVA SA in this work was sufficient enough to induce Q-switching in the laser cavity. It is worth noting that the non-saturable loss of the prepared rGO-Ag film is significantly high, resulting in higher cavity loss and thus favouring the operation of Q-switching instead of mode-locking. Generally, for mode-locking, the number of layers in the SA should be minimal and the SA should be free from impurities. Therefore the proposed SA in this cavity could be thicker with some impurities, affecting the total cavity loss and supressing mode-locking in the cavity.

## 1.0 μm Q-switched YDFL with rGO-Ag/PVA film as Saturable Absorber

### Experimental Setup

**Figure 2:**
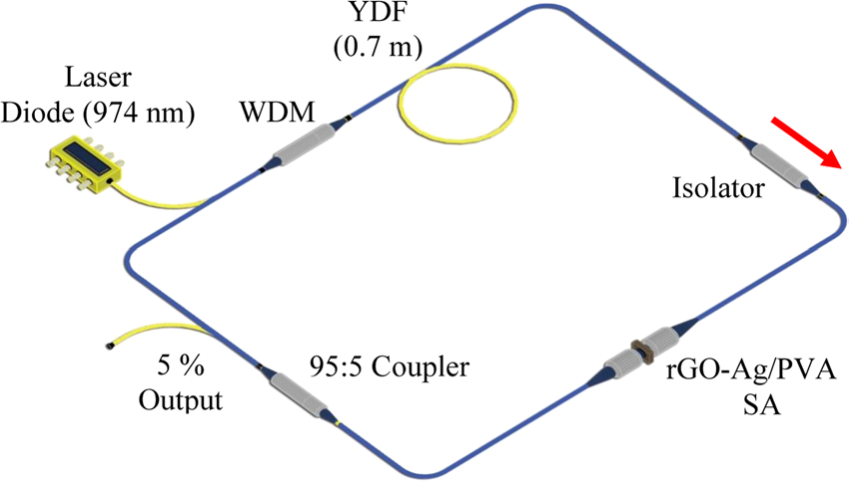
Passively Q-switched YDFL configuration with the insertion of rGO-Ag/PVA SA.

The experimental setup of the Q-switched YDFL with the rGO-Ag/PVA SA is illustrated in Fig. 2. The laser consists of a 0.7 m long YDF, a 95:5 output coupler, an optical isolator (ISO), the rGO-Ag/PVA film SA, a 974 nm laser diode (LD) acting as the pump source and a 980/1060 nm wavelength-division multiplexer (WDM). The total cavity length was measured to be 13.0 m consisting of 12.3 m of HI-1060 fiber and 0.7 m of YDF. The estimated dispersion parameter of the HI-1060 was calculated to be −38.9 ps nm^−1^ km^−1^, while for the YDF was −38 ps nm^−1^ km^−1^. The HI-1060 and YDF have estimated group velocity dispersions (GVDs) of +22.0 ps^2^/km and +21.4 ps^2^/km, respectively at 1.0 μm. The net dispersion of the proposed laser was about +0.2856 ps^2^. The laser output power was measured by a ThorLabs PM100USB optical power meter (OPM), while the optical spectrum was analyzed using a Yokogawa AQ6370C optical spectrum analyzer (OSA) and a Yokogawa DLM2054 oscilloscope (OSC). Measurements in the frequency domain were made using an Anritsu MS2683A radio frequency spectrum analyzer (RFSA).

### Results and Discussion

**Figure 3:**
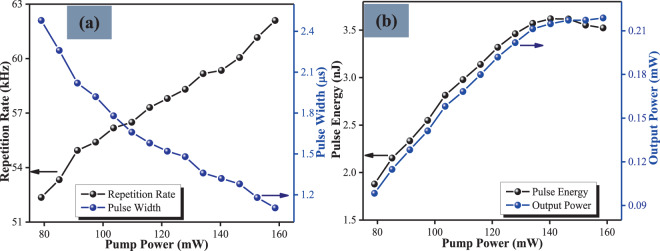
Performance of the passively Q-switched EDF laser at different pumping powers: (**a**) repetition rate and pulse width, and (**b**) pulse energy and output power versus pump power.

Q-switching was observed at a threshold pump power of 72.76 mW in the cavity incorporating the rGO-Ag-PVA SA. It is worth noting that no pulses were observed when the SA was removed, clearly indicating the role of the SA in generating the Q-switched output. As the pump power was increased to 78.97 mW, the pulses become stable. Figure 3 shows the passively Q-switched fiber laser output at different pump powers. The repetition rate and pulse width versus pump power under passively Q-switched operations with the utilization of rGO-Ag-PVA film as SA is shown in Fig. 3(a). It can be observed that the repetition rate showed an incresing trend from 52.33 kHz to 62.10 kHz when the pump power was increased from 78.97 mW to 158.60 mW. In contrast, the individual pulse width showed a decreasing trend from 2.48 μs to 1.10 μs over the same pump power range. This clearly shows the dependency of the repetition rate and pulse width on the pump power and is the normal behaviour of Q-switched pulses. Figure 3(b) shows the relationship between the pulse energy and output power of the Q-switched laser with the pump power. As can be seen, when the pump power was varied from 78.97 mW to 158.60 mW both the pulse energy and output power rose from 1.88 nJ to 3.52 nJ and from 0.10 mW to 0.22 mW respectively. However, the pulse energy shows a slightly decreasing pattern when the pump power exceeded 140.3 mW due to inadequate time for the rGO-Ag/PVA SA to fully recover from saturation at high pump powers^41^.

**Figure 4:**
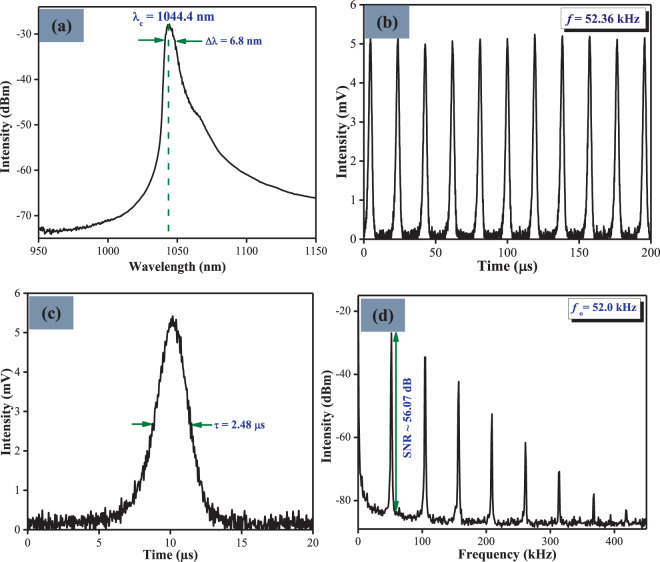
The typical pulse characteristics of the passively Q-switched YDFL taken at the pump power of 78.97 mW: (**a**) optical spectrum, (**b**) pulse train, (**c**) single pulse profile, and (**d**) the RF spectrum.

Figure 4 shows the typical pulse characteristics of the passively Q-switched YDF laser which was recorded at a pump power of 78.97 mW. From the optical spectrum in Fig. 4(a), the generated pulse exhibited a central lasing wavelength at 1044.4 nm with a spectral bandwidth of 6.8 nm. The oscilloscope trace is displayed in Fig. 4(b) and the corresponding single pulse profile is showed in Fig. 4(c). As shown in both figures, the repetition rates of the Q-switched pulses were determined to be 52.33 kHz with a pulse width of 2.48 μs. Figure 4(d) shows the RF spectrum of the pulses where the signal-to-noise ratio (SNR) is measured to be approximately 56.07 dB, thus indicating the stability of the Q-switched pulse laser output.

## 1.5 μm Erbium-Doped Fiber Laser (EDFL) with rGO-Ag/PVA film as Saturable Absorber

### Experimental Setup

**Figure 5:**
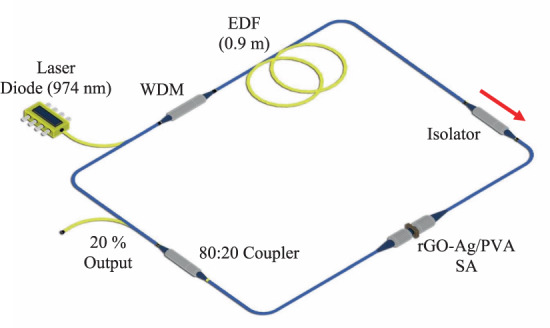
Experimental setup of passively Q-switched EDF laser. LD: laser diode; WDM: wavelength division multiplexer; EDF: Erbium-doped fiber; ISO: isolator; rGO-Ag/PVA SA: reduced graphene oxide-silver nanocomposite/polyvinyl alcohol SA.

Figure 5 illustrates the schematic diagram for the passively Q-switched EDF laser with the rGO-Ag/PVA SA in the laser cavity. The laser cavity was formed by a 974 nm LD as the pump source, a 0.9 m long erbium-doped fiber (EDF) as the gain medium, a 980/1550 nm WDM, an ISO to ensure the unidirectional light propagation, and a 80:20 coupler. The total cavity length is measured to be 11.0 m consisting of 10.1 m of single-mode fiber (SMF-28) and 0.9 m of EDF. The estimated dispersion parameter of the SMF and EDF were calculated to be 18 ps nm^−1^ km^−1^ and -18 ps nm^−1^ km^−1^ respectively. The SMF and EDF have estimated group velocity dispersions (GVDs) values of −23.3 ps^2^/km and +23.2 ps^2^/km, respectively at 1.5 μm. The net dispersion of the proposed laser was about −0.2145 ps^2^. The output of the laser cavity was analysed using a Yokogawa AQ6370C OSA and a Yokogawa DLM2054 OSC together with a 50:50 coupler. An Anritsu MS2683A RFSA and PM100USB Thorlab OPM were also used to measure the SNR and the output power of the Q-switched pulses respectively.

### Results and Discussion

**Figure 6:**
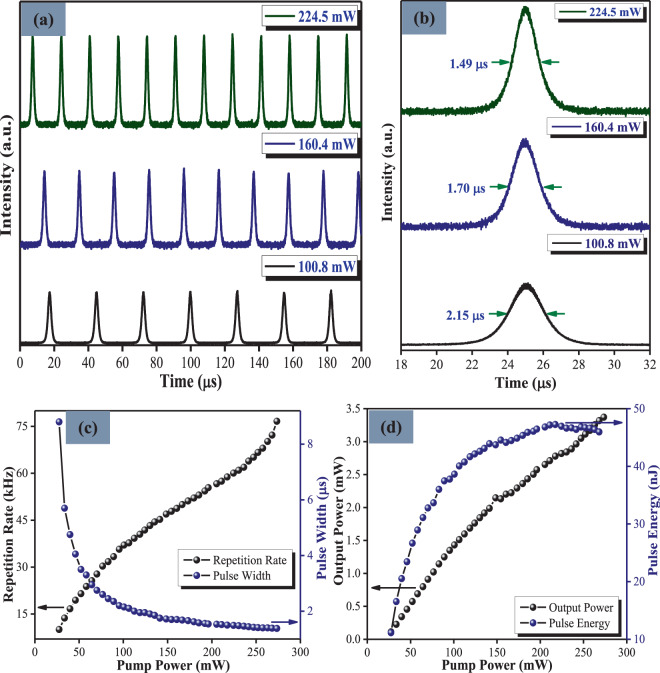
(**a**) Typical output pulse train and (**b**) its corresponding single pulse envelope recorded at different pump power. (**c**) Repetition rate and pulse width and (**d**) output power and pulse energy versus pump power.

The threshold pump power for CW lasing and Q-switching in this cavity was observed to be 18.63 mW and 23.50 mW respectively. At a pump power of 27.42 mW, it was observed that the Q-switched pulses were stable. As before, no Q-switched pulses were observed when the SA was removed from the laser cavity, which clearly indicates the role of the rGO-Ag/PVA SA in generating the pulsed output. The experimental results of the passively Q-switched fiber laser at different pump powers are shown in Fig. 6. Figure 6(a) shows the pulse trains obtained at different pump powers. As can be seen, the generated output pulses have very stable amplitudes at these pumping powers with minimal amplitude fluctuations. The corresponding single pulse profiles are shown in Fig. 6(b). From the experiment, it was observed that the pulse width becomes narrower and the repetition rate increases as the pump power was raised. This is typical Q-switched behaviour in EDF laser cavities^43,44^.

The variation of repetition rates and pulse widths with respect to the different pump powers were measured and the results presented in Fig. 6(c). The repetition rate increased almost linearly from 10.05 kHz to 76.63 kHz as the pump power was increased from 27.42 mW to 273.50 mW. A pulse width of 8.80 μs was obtained under the pump power of 27.42 mW and decreases exponentially to 1.38 μs at a pump power of 273.50 mW. The dependency of the output power and pulse energy on pump power is shown in Fig. 6(d). Within the pump power range of 27.42 mW to 273.50 mW, the output power increased almost linearly from 0.12 mW to 3.32 mW. The pulse energy was measured to range between 11.14 nJ to 44 nJ from the minimum to maximum pump powers.

**Figure 7:**
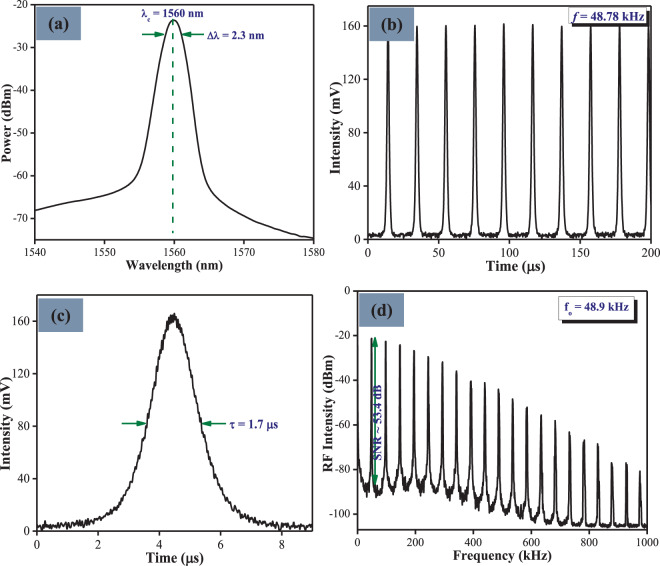
The typical pulse characteristics of the Q-switched EDF laser recorded at pump power of 160.4 mW, showing (**a**) the optical spectrum, (**b**) the oscilloscope trace, (**c**) the single pulse profile, and (**d**) the RF spectrum.

The characteristics of the Q-switched pulses at a pump power of 160.4 mW are given in Fig. 7. As can be seen in Fig. 7(a), the generated pulses retained a symmetrical Gaussian profile with a peak wavelength of 1560 nm and a spectral bandwidth of 2.3 nm. Figure 7(b) shows the pulse train of the Q-switched pulse output with a repetition rate of 48.78 kHz. The single pulse profile at pump power 160.4 mW has a pulse duration of 1.70 μs as observed in Fig. 7(c). From the graph of Fig. 7(d), an SNR value of 53.4 dB can be calculated from the broadband radio frequency (RF) spectrum taken at a 300 Hz resolution bandwidth. As observed, only the fundamental frequency and its harmonics can be seen, with no other frequency components present. This shows that the generated Q-switched pulse of the EDF laser is very stable.

**Figure 8:**
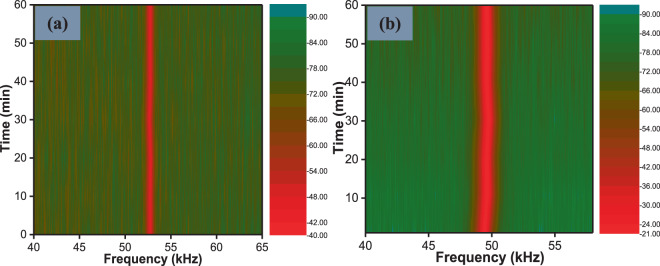
RF spectra obtained within 60 minutes for: **(a)** passively Q-switched YDF laser at the pump power of 78.97 mW and **(b)** passively Q-switched EDF laser at 160.4 mW.

Figure 8 shows the 2D contour maps interpreted from the RF spectra obtained at a fixed pump power of 78.97 mW for the YDF laser and a fixed pump power of 160.4 mW for the EDF lasers. These are shown in Fig. 8(a,b). The stability studies were conducted by continuously running the laser for 1 hour and the RF spectra collected every 10 minutes. It is worth noting that there was no significant drift in the frequency for both lasers.

The performance comparison of passively Q-switched fiber lasers operating at the 1.0 µm and 1.5 µm regions using different materials as SAs has been summarized in Table 1.

**Table 1:** Performance comparison of passively Q-switched fiber laser operating at 1.0 µm and 1.5 µm region using different types of SA materials.

Gain Medium	SA	Operating Wavelength (nm)	Pump Power Range (mW)	Minimum Pulse Width (μs)	Maximum Repetition Rate (kHz)	Maximum Pulse Energy (nJ)	Ref.
YDF	ZnO	1038	115.20–196	1.60	50	2.80	^45^
	CuNP PVA	1040.60	157–229	3.89	104.30	7.48	^46^
	Co_3_O_4_	1043.64	144.40–165.40	4	86.66	154	^47^
	GO	1074.59	167–470	2.66	58.95	2980	^48^
	Ag nanoplate	1062	145–345	1.84	65.70	53.10	^49^
	**rGO-Ag/PVA**	**1044.40**	**78.97–158.60**	**1.10**	**62.10**	**3.52**	**This work**
EDF	BP	1562.87	50–195	10.32	15.78	94.3	^18^
	rGO/FM	1560	50–170	3.53	47.04	48.19	^50^
	AgNP/PVA	1560	140.8–236.20	8.16	55.70	34.70	^51^
	CVD Graphene	~ 1500	57.20–114.40	3.20	53.20	17.41	^52^
	**rGO-Ag/PVA**	**1560**	**27.42–273.50**	**1.38**	**76.63**	**44**	**This work**

FM = Fluorine mica, CVD = Chemical vapour deposition; GO = Graphene oxide; Ag = Silver; ZnO = Zinc oxide; BP = Black phosphorus; Co3O4 = Cobalt oxide; CuNP = Copper nanoparticles.

In ref. ^48^, the measured pulse energy was about 2980 nJ at a pump power of 470 mW with an operating wavelength of 1074.59 nm using GO as an SA. Meanwhile, ref. ^49^ used Ag nanoplates as an SA to generate pulses with a maximum energy of 53.10 nJ at 345 mW and an operating wavelength of 1062 nm. Even though both works could obtain higher pulse energies compared to this work, however the proposed YDF laser was able to generate Q-switched pulses at a much lower pump power with a narrower pulse width. On top of this, this work also has a better output performance in terms of lower pump power and the pulse width is narrower when compared to refs. ^45–47^. In ref. ^50^, Q-switched pulses were produced at 1560 nm with a maximum pulse energy and repetition rate of 48.19 nJ and 47.04 kHz respectively using rGO as an SA. The maximum pulse energy obtained is slightly higher than that of this work, however, the pulse width of this design’s output is much narrower. Moreover, the repetition rate of the demonstrated Q-switched EDF laser attained in this work is higher than those in refs. ^18,50–52^. Overall, the performance of the passively Q-switched EDF laser using the rGO-Ag/PVA SA of this work is better than those given in the table.

## Methods

### Chemicals and reagents

The graphite flakes used in this work to form the rGO nanoparticles were obtained from Asbury Graphite Mills. 85% Phosphoric acid (H_3_PO_4_), 98% sulfuric acid (H_2_SO_4_), 37% hydrochloric acid (HCl and 25% ammonia solution (NH_4_OH) were obtained from Merck. The 99.9% Silver nitrate (AgNO_3_), 30% hydrogen peroxide (H_2_O_2_) and polyvinyl alcohol (PVA) with MW ~31,000 were obtained from Sigma Aldrich. Potassium permanganate (KMnO_4_) was purchased from R&M Chemicals. All chemicals were used as received without any further processing.

### Preparation of rGO-Ag Nanocomposite

Initially, graphene oxide (GO) was synthesized using the simplified Hummers’ method. This method is a commonly used method, and has been described in detail in ref. ^53^. 10 mL of GO solution with the concentration of 1 mg/mL was sonicated for 15 min to obtain a homogeneous dispersion. Then, 2 mL of AgNO_3_ solution was added into the GO solution and was stirred for 15 minutes. Next, 13 mL of NH_4_OH solution was injected into the mixture while stirring at room temperature for another 15 minutes. Subsequently, the mixture was transferred into a Teflon-lined stainless steel autoclave and heated in an oven at 180 °C for 16 hours. After the hydrothermal reaction was completed, the resulting product was collected by centrifugation at 6000 rpm for 15 minutes. The remaining black precipitate was then washed with deionized water (DIW) and ethanol several times and dried overnight at 60 °C. The reference samples of rGO and AgNPs were prepared according to the technique described above, but without the addition of the AgNO_3_ and GO solutions respectively.

### Fabrication of rGO/Ag-PVA Film

A solution casting technique was used to fabricate the rGO-Ag/PVA film. In this process, polyvinyl alcohol (PVA) polymer was employed as the host film. Initially, 100 mg of the PVA powder was mixed with 10 ml of deionized (DI) water and the solution was stirred at 60 °C for 2 hours. 10 mg/mL of rGO-Ag solution was prepared concurrently by dissolving 100 mg of rGO-Ag powder in 10 mL of DI water under bath sonication for 30 minutes. Next, the PVA solution and rGO-Ag solution was added together into a beaker and the mixture was stirred continuously at 60 °C on the hot plate. This process was continued until only 1/3 of the mixture remained. The remaining mixture was poured onto a petri dish and the solvent allowed to evaporate in an oven at 60 °C for 2 hours. The film was allowed to cool down to room temperature and peeled from the petri dish afterward and labelled as rGO-Ag/PVA film.

## Conclusion

This work has demonstrated stable passively Q-switched YDF and EDF lasers using rGO-Ag/PVA films as SAs operating at 1044.4 nm and 1560 nm respectively. This was the first time that Q-switched operation in YDF and EDF lasers has been achieved using an rGO-Ag/PVA SA. In the YDF laser, the highest pulse repetition rate of 62.10 kHz and shortest pulse width of 1.10 µs were observed at a maximum pump power of 158.60 mW. The generated pulses exhibited the highest pulse energy of 3.52 nJ with a maximum output power of 0.22 mW. In addition, a maximum repetition rate of 76.63 kHz and minimum pulse width of 1.38 μs were obtained at the maximum pump power of 273.50 mW for the EDF laser. The generated pulse yields its highest pulse energy of 44 nJ with a maximum output power of 3.32 mW. The stability of the Q-switched YDF and EDF lasers were verified with the SNR values of 56.07 dB and 53.4 dB at pump powers of 78.97 mW and 160.4 mW respectively. The combination of the AgNPs with rGO has helped to increase the performance of the passively Q-switched fiber laser operating at the 1.0 and 1.5 μm regions. The rGO-Ag/PVA film exhibits promising potential as an SA for use in various photonic applications.
